# Metabolic role of dipeptidyl peptidase 4 (DPP4) in primary human (pre)adipocytes

**DOI:** 10.1038/srep23074

**Published:** 2016-03-17

**Authors:** Pia Zilleßen, Jennifer Celner, Anita Kretschmann, Alexander Pfeifer, Kurt Racké, Peter Mayer

**Affiliations:** 1 Federal Institute for Drugs and Medical Devices (BfArM), 53175 Bonn, Germany; 2 Institute for Pharmacology and Toxicology, University of Bonn, 53105 Bonn, Germany

## Abstract

Dipeptidyl peptidase 4 (DPP4) is the target of the gliptins, a recent class of oral antidiabetics. DPP4 (also called CD26) was previously characterized in immune cells but also has important metabolic functions which are not yet fully understood. Thus, we investigated the function of DPP4 in human white preadipocytes and adipocytes. We found that both cell types express DPP4 in high amounts; DPP4 release markedly increased during differentiation. In preadipocytes, lentiviral DPP4 knockdown caused significant changes in gene expression as determined by whole-genome DNA-array analysis. Metabolic genes were increased, e.g. PDK4 18-fold and PPARγC1α (=PGC1α) 6-fold, and proliferation-related genes were decreased (e.g. FGF7 5-fold). These effects, contributing to differentiation, were not inhibited by the PPARγ antagonist T0070907. Vice versa, the PPARγ agonist pioglitazone induced a different set of genes (mainly FABP4). DPP4 knockdown also affected growth factor signaling and, accordingly, retarded preadipocyte proliferation. In particular, basal and insulin-induced ERK activation (but not Akt activation) was markedly diminished (by around 60%). This indicates that DPP4 knockdown contributes to adipocyte maturation by mimicking growth factor withdrawal, an early step in fat cell differentiation. In mature adipocytes, DPP4 becomes liberated so that adipose tissue may constitute a relevant source of circulating DPP4.

Diabetes mellitus is a major health problem in the 21^st^ century world-wide and hence, there is a growing line of antidiabetic drugs and therapies^1^,^2^. Gliptins, small molecular inhibitors of the peptidase DPP4 (dipeptidyl peptidase 4), were developed recently as a new class of antidiabetic drugs. DPP4 degrades incretin peptides (e.g. GLP1) and is widely known for its regulatory effect in glucose metabolism^3^,^4^. Findings in DPP4 knockout mice revealed that absence of this enzyme improves glycemic control and leads to reduced fat mass^5^,^6^. This made DPP4 inhibitors promising candidates for treating human Type 2 diabetes. Inhibition of the enzymatic activity of DPP4 prevents the rapid cleavage of incretins and as a consequence, higher endogenous incretin levels are obtained which enhance the glucose-induced insulin secretion. This so-called incretin-like effect is responsible for approximately 60% of postprandial insulin secretion^7^. DPP4 is a ubiquitous glycoprotein and occurs as a cell membrane bound protein as well as in a soluble, extracellular form^8^,^9^. It is well established that DPP4 has multiple functions^8^,^10^, which raises concerns of unexpected off-target effects of gliptins. Changes in the peptidase activity or enzyme concentration were described to be associated among others with obesity, diabetes and neurologic and inflammatory diseases^9^,^11^,^12^. Beside of cleaving several substrates (e.g. chemokines, neuropeptides, growth factors^9^,^10^,^13^), there is evidence of non-enzymatic functions by interaction with ADA (adenosine deaminase) and other matrix proteins or by immunological mechanisms^8^,^14^,^15^. In addition, DPP4 was originally identified as CD26 antigen on lymphocytes so that an immunological role of this gene product was assumed^16^. Therefore, concerns about potential unintended immunosuppressive effects were expressed. Meanwhile, DPP4 is discussed as a potential link between obesity and the metabolic syndrome or insulin resistance^17^,^18^,^19^. DPP4 was suggested to be a new adipokine, involving the adipose tissue as a possible source for the circulating DPP4 activity in the plasma, which is relevant for GLP1 degradation, suggesting that DPP4/CD26 could play a major role in metabolism.

The adipose tissue as a major endocrine and energy storage organ plays an important role in metabolic systems and insulin action^20^,^21^,^22^ and is already a target for another class of antidiabetics, the glitazones. The latter, being PPARγ-agonists, lead to enhanced insulin sensitivity, accompanied by an increased differentiation and fat accumulation. However, the role of DPP4 in human adipose tissue is currently unclear. Furthermore, recent animal studies with DPP4 inhibitors support the notion that DPP4 may play a functional role within adipose tissue, because DPP4 inhibition has been seen to prevent adipose tissue inflammation and development of glucose intolerance in high fat diet induced obesity in mice^23^. Based on these considerations, we aimed to gain more insight in the role of DPP4 in primary cultured human white preadipocytes and adipocytes.

## Results

### Expression and release of DPP4 in human white (pre)adipocytes

**Figure 1:**
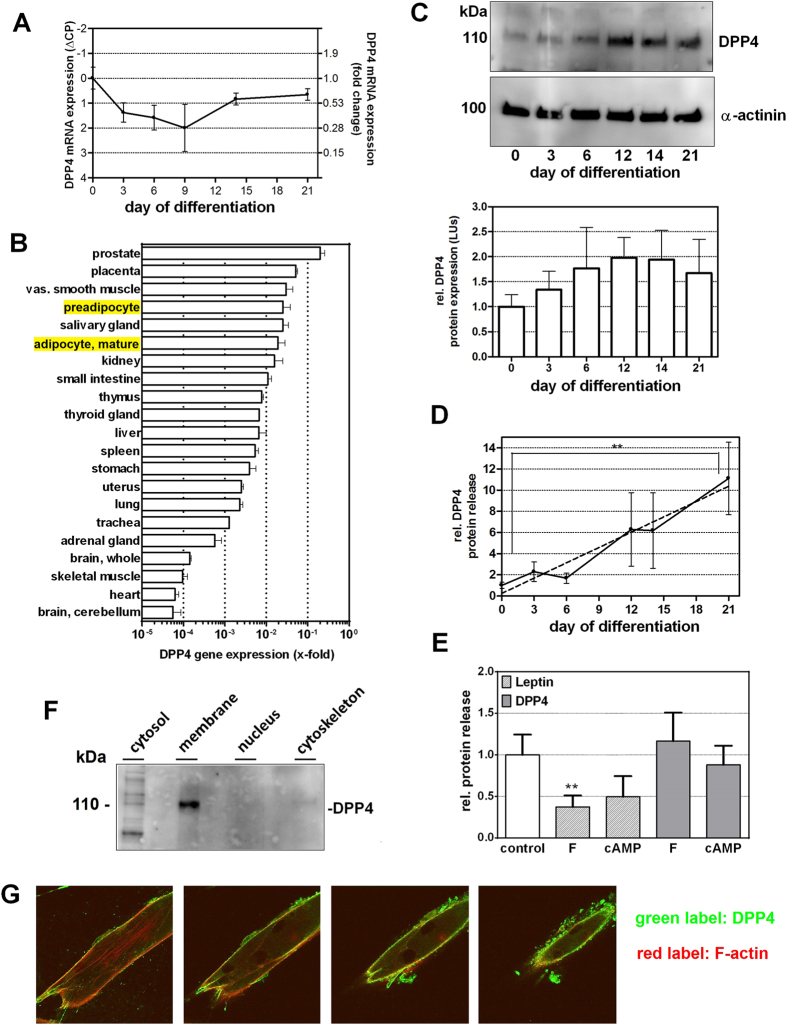
DPP4 expression, release and localization in human white (pre)adipocytes. Part (**A**) Time course of DPP4 expression was measured by RT PCR during differentiation. CP values were normalized to the housekeeping gene GAPDH, and are presented relative to Day 0. The left y-axis shows the original ΔCP values, and the calculated fold change vs. Day 0 is displayed on the right y-axis. Data are displayed as mean values ± SEM, n = 4. Part (**B**) DPP4 expression was measured by Real Time PCR in different human tissues (RNA Panel from Clontech, Mountain View, CA, USA), normalized to the housekeeping gene GAPDH, and is presented as mean fold change values + SEM, n ≥ 2, in a logarithmic scale. Part (**C**) DPP4 protein levels during differentiation were analyzed by Western blotting followed by densitometric quantification. The upper panel shows a representative blot, which was cropped for clarity. The full gel is shown in Supplementary Figure S2A. For quantification, at least three blots per time point were used. Densitometric data were normalized to α-actinin and are displayed relative to Day 0 in the lower panel as mean values + SEM. Part (**D**) DPP4 release during differentiation was assessed by ELISA. Data are represented relative to Day 0 as mean values ± SEM (full line), n = 5. Statistical evaluation was done by regression analysis, **p < 0.01. The regression line is shown dashed. Part (**E**) Effect of lipolysis, induced by forskolin (10 μM, “F”) or DBcAMP (100 μM, “cAMP”) on the liberation of DPP4 and leptin into the cell culture supernatants over 24 hours. Data are presented relative to untreated control as mean values ± SEM, n ≥ 4. Statistical analysis was done by one sample t test; **p < 0.01 vs. control. Part (**F**) Presence of DPP4 in different cell fractions as indicated was analyzed by Western blotting after cell fractionation; the blot was cropped for clarity; the whole gel is shown in Supplementary Figure S2B. Part (**G**) Microscopic images (40x) showing immunofluorescence staining of DPP4 (green) to determine its subcellular localization. The cytoskeleton (F-actin) is labeled in red.

In order to examine the role of DPP4 in the human adipose tissue, we first measured the expression of this enzyme in primary human white adipocytes at mRNA and protein level during differentiation. Mature adipocytes were obtained by *in-vitro* differentiation of primary cultured preadipocytes, following the protocol described in the Methods section. DPP4 mRNA was expressed in preadipocytes (i.e. Day 0 of differentiation) and mature adipocytes (up to Day 21 of differentiation) at comparable levels (Fig. 1A). There was a brief dip in DPP4 expression at Days 3 to 9 of differentiation (Day 0, switch to differentiation medium) but there were no major long-term changes.

Comparison of DPP4 expression level in preadipocytes and adipocytes with various other human tissues (Fig. 1B) revealed that preadipocytes and mature adipocytes express 4^th^ and 6^th^ highest level of DPP4, respectively (highlighted in Fig. 1B).

DPP4 protein weakly increased with differentiation as revealed by Western blotting and consecutive densitometric quantification (Fig. 1C), but statistical significance was not reached.

In order to test whether (pre)adipocytes could be a source of soluble DPP4, we analyzed cell culture supernatants, collected at different time points of differentiation, by ELISA (Fig. 1D). Preadipocytes liberated DPP4 to a low extent only (around 1 ng/ml), but DPP4 release markedly increased during differentiation. Mature adipocytes released up to 11-times more DPP4 than non-differentiated cells. A linear regression analysis revealed statistical significance. It should be noted that DPP4 is not stored in vesicles before release (instead, the extracellular part of this membrane protein is cleaved off) so that cellular DPP4 content does not need to increase when liberation of DPP4 increases.

In a next step, we questioned which events could influence DPP4 release. Cellular triglyceride content was a candidate because triglyceride storage and liberation is a prominent function of adipocytes. In order to change the triglyceride content of the cultured adipocytes, we induced lipolysis by treatment with the cAMP-mimetics forskolin (10 μM) or DBcAMP (100 μM) for 24 h (Fig. 1E). Triglyceride degradation was verified by measuring the increase of glycerol content in the cell culture supernatants. DPP4 release was measured by ELISA 24 h after induction of lipolysis. Leptin secretion was also determined for comparison. It turned out that leptin secretion was significantly reduced following lipolysis whereas no influence on DPP4 release was detected (Fig. 1E).

For further characterization, we determined the intracellular localization of DPP4 protein in preadipocytes. Western blotting following cell fractionation revealed a strong DPP4 signal in the membrane fraction (Fig. 1F). The cytosolic fraction showed several weak bands, probably due to unspecific binding of the antibody. No signal could be detected in the nucleus and in the cytoskeleton. In accordance with this observation, detection of DPP4 protein in preadipocytes by immunofluorescence with z-stack analysis (Fig. 1G) revealed DPP4 (green signal) localization primarily in the outer cell regions, i.e. in a typical appearance of membrane proteins.

Taken together, DPP4 is expressed in preadipocytes and adipocytes, is located primarily in the cell membrane from which it becomes increasingly released during maturation.

### Gene expression profile after DPP4 knockdown

**Figure 2:**
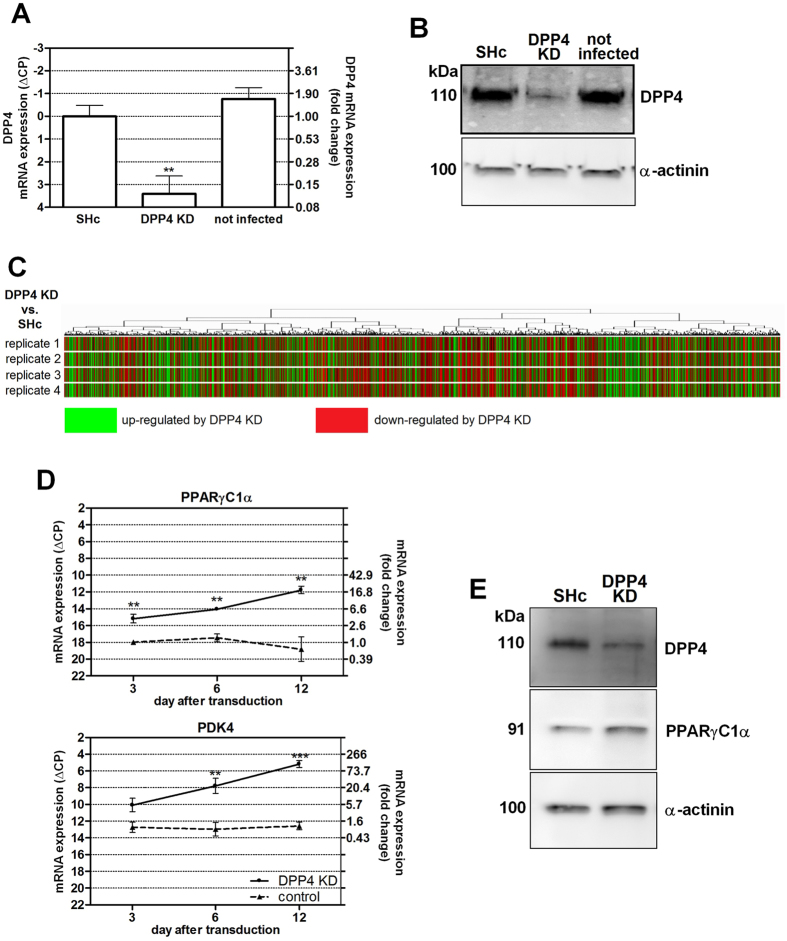
Lentiviral knockdown of DPP4 in human preadipocytes. Human preadipocytes were stably transduced with shRNA constructs directed against DPP4 mRNA by lentiviral vectors. Unspecific, non-target shRNA was used as a negative control (labeled “SHc” in the figure). Knockdown (KD) of DPP4 expression was confirmed on mRNA Part (**A**) and protein Part (**B**) level by quantitative Real Time PCR and Western blotting, respectively. The picture of the Western blot was cropped for clarity. The entire lanes are shown in Supplementary Figure S2C. PCR data are presented as mean ∆CP values (normalized to GAPDH, relative to SHc) ± SEM (left y-axis) and calculated fold change values vs. control (right y-axis), n ≥ 5. Statistical analysis was done by one-way ANOVA with Dunnett post-test; **p < 0.01 vs. negative control. Part (**C**) At least 5-fold changes in gene expression resulting from DPP4 knockdown, measured by whole genome DNA array hybridization, are visualized in a heat plot. Hybridization was performed in two-color mode; each line represents the difference between a DPP4 knockdown and sh-control sample. The four lines represent four biological replicates. Up-regulated genes in DPP4 knockdown compared to control are marked in green, down-regulated genes in red. The color intensity indicates the expression level of the respective gene. Part (**D**) Changes in the expression of two representative genes (PPARγC1α and PDK4) over time after infection were followed by quantitative PCR. Data represent mean ∆CP values (vs. GAPDH) ± SEM (left y-axis) and calculated fold change values vs. control (SHc at Day 0) on the right y-axis, n ≥ 3. Statistical analysis was done by *t* test; *p < 0.05; **p < 0.01 vs. control. Part (**E**) shows a Western blot confirming the up-regulation of PPARγ1Cα on protein level when DPP4 is suppressed. The blot was cropped for clarity. The entire lanes are shown in Supplementary Figure S2D. The results for DPP4, PPARγ1Cα and the loading control α-actinin from cells infected with sh-control vector (“SHc”) and sh-DPP4 vector (“DPP4 KD”), respectively, are shown as indicated in the figure.

As described above, DPP4 was highly expressed in preadipocytes but was hardly released from these cells. This implies a different function of DPP4 in preadipocytes as compared to mature adipocytes. For closer investigation, we produced preadipocytes with a stable knockdown of DPP4 expression, achieved by lentiviral shRNA (see Methods section). Successful lentiviral transduction was verified with a GFP construct (Supplementary Figure S1A). Unspecific effects of the lentiviral vector were excluded by using a non-targeting shRNA (“sh-control” or “SHc”) as negative control in each experiment. Furthermore, two other independent shRNA constructs, also directed against DPP4, were tested and found to induce the same set of genes (Supplementary Figure S1B). Knockdown of DPP4 was verified at mRNA (Fig. 2A) and protein level (Fig. 2B).

No clear hints were available what the function of DPP4 in preadipocytes could be so that we started our study with a screen for changes in gene expression using a whole genome oligo microarray (Agilent). A heat map of four replicate experiments visualizing genes that were altered at least 5-fold is shown in Fig. 2C. Salient genes, altered at least two-fold and forming functional clusters are listed in Table 1. The most pronounced changes were found in genes involved in lipid metabolism (with an up-regulation in most cases) and in some proliferation related genes (down-regulated in most cases) as summarized in Table 1. For example, a 5- to 24-fold up-regulation of FABP4, PDK4 and PPARγC1α was observed.

The transcription factor C/EBPε, a relative of C/EBPα and C/EBPβ, was induced 20-fold. C/EBPα and C/EBPβ are known to be involved in adipocyte differentiation. C/EBPε was initially detected in lymphoid and myeloid cells based on its structural similarity to C/EBPα and C/EBPβ^24^, and is assumed to regulate their differentiation^25^. The function of C/EBPε in preadipocytes is unknown but, due to its structural similarity, C/EBPε could mimic the effects of C/EBPα and/or C/EBPβ. Other transcription factors were also increased, such as members of the KLF family (e.g. KLF15, −5, −2). Among the genes induced or suppressed at least twofold, four functional clusters became obvious (Table 1), namely lipid metabolism, proliferation, structural genes including cell-cell contact and cell migration.

Representative metabolic and proliferative genes were selected for further investigation by quantitative PCR. We studied the time course of expression and the interaction with other agents known to be involved in adipocyte differentiation. The time course of PPARγC1α and PDK4 expression after knockdown is shown in Fig. 2D. A marked increased in expression was already detected on Day 3 (4-fold for PPARγC1α and 5-fold for PDK4), and expression further increased up to 27-fold for PPARγC1α and 116-fold for PDK4 on Day 12. Thus, the effect was persistent over time. Although these experiments showed that the changes in gene expression are stronger at Day 12 after infection than at Day 3, most experiments were performed at Day 3 or Day 4 after infection to avoid the need of puromycin selection (see Methods section), which could introduce unspecific effects.

The gene PPARγC1α, also known as PGC1α, is a transcription regulator and appears to have a crucial role in cellular energy metabolism, in particular in mitochondrial biogenesis^26^. Therefore we confirmed its up-regulation also on the protein level by Western blotting (Fig. 2E).

DPP4 is a multifunctional protein which actions beyond peptidase activity. Thus, we tested whether the observed effects by the knockdown could be due to the peptidase activity. The latter can be inhibited by sitagliptin (10 μM). No effect of sitagliptin on the expression of the genes responsive to DPP4 knockdown was observed (not shown). This indicates that a non-peptidase function of DPP4 is responsible for the observed regulation of gene expression.

### Comparison of DPP4 knockdown with PPARγ effects

**Figure 3:**
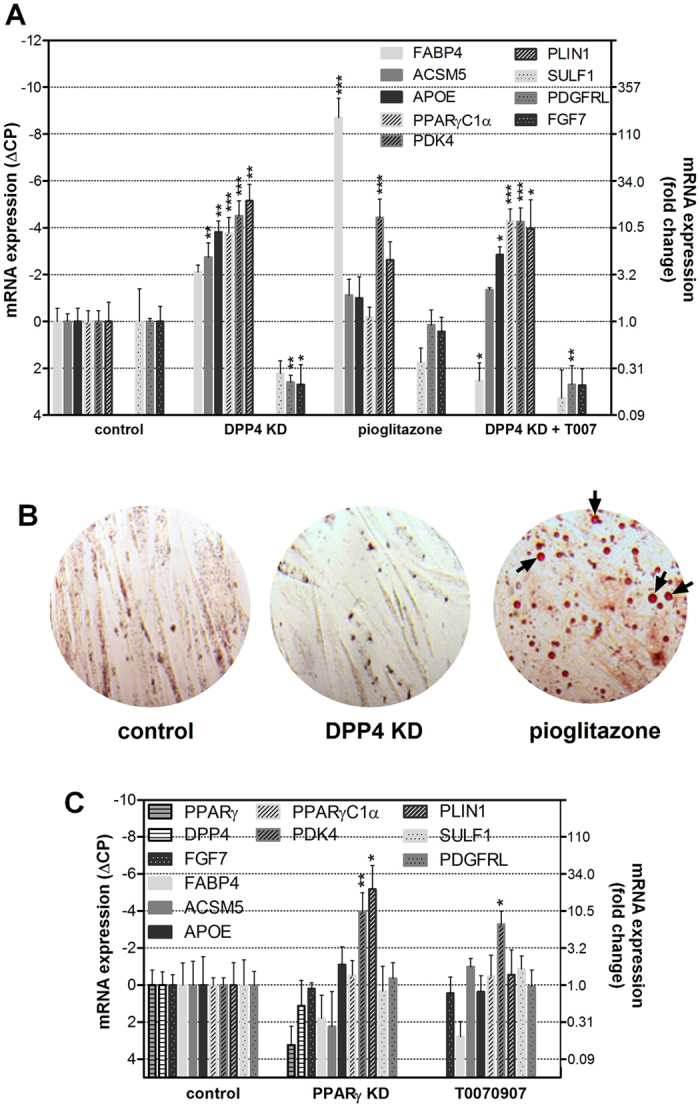
Effects of DPP4 knockdown compared to the effects of pioglitazone. Part (**A**) Human preadipocytes were stable transduced by lentiviral shRNA constructs against DPP4 or were incubated with a PPARγ agonist (pioglitazone, 10 μM) for 3 days. Incubation of DPP4 knockdown cells with a PPARγ inhibitor (T0070907, 10 μM) is shown on the right. Expression of representative genes was analyzed by quantitative Real Time PCR. Values were normalized to the housekeeping gene GAPDH, and are presented relative to control cells infected with non-targeting shRNA. The left y-axis shows the original ΔCP values, and the calculated fold change vs. Day 0 is displayed on the right y-axis. Part (**B**) shows Oil Red O staining of lipids (red) in human preadipocytes after DPP4 knockdown (“DPP4 KD”, mid panel) or after treatment with pioglitazone (10 μM, right panel) for 10 days. Exemplary lipid vacuoles are marked by arrows. Control cells treated with non-targeting shRNA are shown in the left panel. Part (**C**) Gene expression was measured by RT PCR in human preadipocytes after lentiviral transduction with a shRNA construct against PPARγ (“PPARγ KD”) or after treatment with the PPARγ inhibitor T0070907. Representation of data is the same as in Part (**A**). In Parts (**A**) and (**C**), bars represent mean values + SEM, n ≥ 4, Statistical analysis was done by one-way ANOVA with Dunnett post-test; *p < 0.05; **p < 0.01; ***p < 0.001 vs. control.

The alterations in gene expression induced by DPP4 knockdown imply that the latter contributes to the differentiation of the preadipocytes as outlined above. A well-established inducer of adipocyte differentiation is the transcription factor PPARγ^27^. Therefore, we asked whether the effects of DPP4 knockdown described above could be mediated by PPARγ. We compared the effects of DPP4 knockdown with the effects of treatment with the PPARγ agonist pioglitazone (100 μM for 72 h, Fig. 3A). The sets of genes that were induced by DPP4 knockdown and pioglitazone treatment, respectively, were similar since both sets were indicative for adipocyte differentiation. However, there were marked differences. A main feature of the pioglitazone effect was the strong induction of FABP4. With DPP4 knockdown, FABP4 induction was much weaker. In contrast, DPP4 knockdown strongly and consistently induced PPARγC1α and APOE which were hardly affected by pioglitazone (Fig. 3A). Furthermore, expression of the growth factor FGF7 was suppressed by DPP4 knockdown but was not affected by pioglitazone. For further confirmation of independent actions of DPP4 and PPARγ, we employed the PPARγ antagonist T0070907 (10 μM). The changes in gene expression were similar with DPP4 knockdown alone and with DPP4 knockdown plus T0070907 (Fig. 3A). Thus, T0070907 did not alter the gene expression induced by DPP4 knockdown.

A further difference between the action of DPP4 knockdown and PPARγ activation was revealed by determining lipid accumulation. Lipid droplets (marked by arrows in the figure) were observed after pioglitazone stimulation but not after DPP4 knockdown (Fig. 3B).

T0070907 alone had no major effects on the DPP4 knockdown expression profile except for some up-regulation of PDK4 (Fig. 3C, right group of bars). In order to confirm that T0070907 had no unspecific effects (i.e. actions beyond PPARγ inhibition) we compared the effect of T0070907 on gene expression with the effect of PPARγ knockdown (Fig. 3C, mid group of bars). Overall, the pattern was similar except for an up-regulation of PLIN1 by PPARγ knockdown but not by T0070907 treatment.

### Effect of DPP4 knockdown in later stages of differentiation

**Figure 4:**
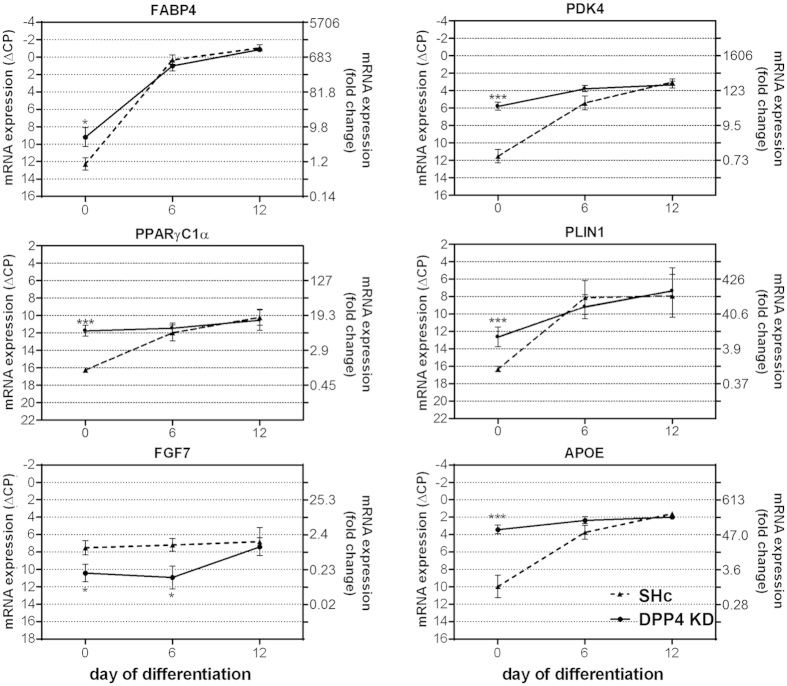
Effect of DPP4 knockdown during differentiation. Human preadipocytes were stable transduced by lentiviral shRNA constructs against DPP4; infected cells were selected with puromycin and differentiated for up to 12 days. Gene expression was measured by Real Time PCR and was normalized to the housekeeping gene GAPDH. Full lines represent the time course of gene expression during differentiation in DPP4 knockdown (“DPP4 KD”) cells, broken lines refer to cells transduced with non-targeting shRNA (sh-control, “SHc”). Data are displayed as mean ∆CP values ± SEM (left y-axis). For better understanding, calculated values of fold change vs. control (SHc at Day 0) are indicated on the right y-axis. Statistical analysis (n ≥ 3) was done by *t* test; *p < 0.05; ***p < 0.001 vs. control.

DPP4 knockdown elicited changes in gene expression in preadipocytes. Thus, we investigated the effect of DPP4 knockdown also in later stages of adipocyte maturation. Preadipocytes stably transduced with DPP4 shRNA were differentiated according to the standard protocol (see Methods section), and the expression of the genes of interest was studied by RT-PCR at Days 0, 6 and 12 of differentiation (Fig. 4). For comparison, the differentiation protocol was also performed with cells expressing non-target control shRNA.

Compared to Day 0 of differentiation, the effect of DPP4 knockdown diminished during differentiation despite DPP4 mRNA remained suppressed. At Day 0 of differentiation (i.e. before switching the cells to differentiation medium), the expression rates of the genes shown were higher (or lower in case of FGF7) in DPP4 knockdown cells compared to sh-control cells. The differentiation process caused an increase of the metabolic genes (FABP4, PDK4, PPARγC1α, PLIN1 and APOE). This effect was more pronounced in the sh-control cells so that after differentiation (Day 12) the expression levels of these metabolic genes were virtually identical in the DPP4 knockdown and in the sh-control cells (Fig. 4). The growth factor FGF7 was decreased in preadipocytes in response DPP4 knockdown, but also for this gene the expression levels became similar in DPP4 knockdown and sh-control cells after differentiation (Fig. 4).

### Effect of DPP4 knockdown on intracellular signaling

**Figure 5:**
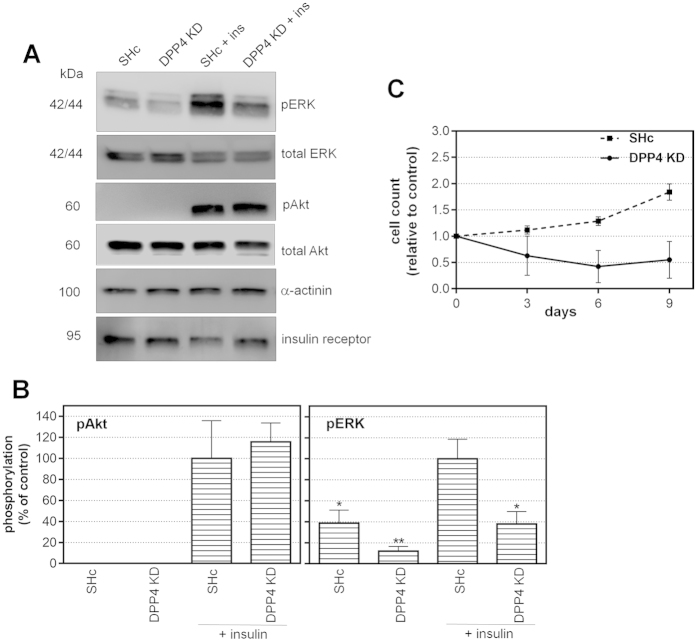
Effect of DPP4 knockdown on ERK and Akt activity. Human preadipocytes were transduced by lentiviral shRNA directed against DPP4 (labeled “DPP4-KD” in the figure) or, as control, by non-targeting shRNA (labeled “SHc” in the figure. Part (**A**) Activation of signaling pathways was analyzed by Western blotting with antibodies directed against the phosphorylated (active) form of the respective signaling protein, pERK (phospho-Extracellular-signal Regulated Kinase) or pAkt (phospho-Akt1). The effect of DPP4 knockdown on insulin (ins) signaling via the pAkt and the pERK pathway is shown. The insulin concentration used was 100 nM, incubation time was 10 min. Detection of α-actinin served as loading control. Insulin receptor expression was also detected in the preadipocytes (lower panel of Part A) and was not affected by treatment. The blots were cropped for clarity. Uncropped pictures are shown in Supplementary Figure S3. The densitometric quantification of the phosphoproteins is shown in Part (**B**). Statistical analysis was done by one-way ANOVA with Dunnett post-test; *p < 0.05; **p < 0.01 vs. insulin-treated sh-control (SHc). Part (**C**) Proliferation of the preadipocytes after DPP4 knockdown vs. SHc was assessed by cell counting at various time points as indicated.

For closer investigation of the mechanisms by which DPP4 knockdown exerts the described effects on gene expression, we studied the activation of protein kinase signaling pathways (Fig. 5). Growth factor withdrawal is the first step of adipocyte differentiation *in vitro*, and it was reported that *in vivo* an autocrine EGF-related growth factor, Pref-1, prevents differentiation via activation of ERK^28^,^29^. In cultured preadipocytes we observed a basal activity of the ERK pathway, measured as phosphorylated ERK (pERK) by Western blotting. Preadipocytes express insulin receptors (Fig. 5A); insulin receptor expression was not influenced by DPP4 knockdown. ERK phosphorylation was markedly enhanced by stimulation with insulin (100 nM for 10 min). After knockdown of DPP4, insulin-induced ERK phosphorylation was significantly weaker (Fig. 5A; densitometric quantification in Fig. 5B). In contrast, activation of the pAkt pathway by insulin was not diminished after DPP4 knockdown (Fig. 5A,B). Thus, DPP4 knockdown selectively attenuated the growth factor-like signaling of insulin.

In line with the described effects on growth factor signaling, DPP4 knockdown prevented further proliferation of the preadipocytes as measured by cell counting over time (Fig. 5C).

Taken together, DPP4 knockdown in preadipocytes diminished the ability of insulin and probably other growth factors to activate the ERK signaling pathway. This mimics growth factor withdrawal, leads to growth arrest and could thereby contribute to initiate the first step of differentiation.

## Discussion

The various functions of DPP4 have been widely discussed, among others in the fields of immunology, (neuro-)endocrinology and glucose homeostasis^8^,^10^. However, the role of DPP4 in human adipose tissue is still unclear. Our results now revealed a strong expression of this gene in human white preadipocytes and adipocytes and revealed a possible contribution of DPP4 to the adipocyte differentiation process. Furthermore, mature adipocytes were identified as a potential source of circulating DPP4.

Adipocyte maturation is a complex process and involves several different mediators and signaling pathways^30^,^31^,^32^,^33^. Among these are the two master regulators PPARγ and the C/EBP family^32^,^34^. Our knockdown experiments revealed changes in the expression of functional gene clusters indicative for adipocyte differentiation.

Investigation of signaling pathways identified a potential mechanism by which DPP4 knockdown could contribute to differentiation. It became obvious that basal and insulin-induced ERK phosphorylation was attenuated. In contrast, activation of the Akt pathway by insulin was not affected, arguing for a selective action of DPP4 on growth factor signaling via ERK.

It should be noted that insulin probably has a dual role in respect to adipocyte differentiation^32^. On one hand, insulin promotes differentiation and is a component of the differentiation medium. For this effect activation of the pAkt signaling pathway appears to be relevant. On the other hand, by activation of ERK insulin behaves like a growth factor and may thereby counteract the onset of differentiation. The role of ERK in adipocyte differentiation is not fully clear^32^, but in the case of the EGF-related growth factor Pref-1, which acts on preadipocytes in an autocrine way, it was clearly shown that ERK activation by Pref-1 prevents differentiation^28^,^29^.

The effects of DPP4 knock-down were not influenced by inhibition of PPARγ, an important player in adipocyte maturation but acting at a later stage of this process. Accordingly, the set of genes induced by DPP4 knockdown differed from the set induced by PPARγ. The action of DPP4 at an early step in the differentiation process also explains why DPP4 knockdown, in contrast to PPARγ activation, did not promote triglyceride accumulation because the latter most likely is a late event in adipocyte maturation.

Beside of metabolic genes, genes encoding extracellular matrix proteins and proteins being involved in cell-cell interaction and migration were altered by DPP4 knockdown. A link between the extracellular matrix composition and the differentiation competence involving the action of matrix metalloproteinases (MMPs) was described already^33^. Moreover, a role for DPP4 in the modulation of the extracellular matrix could be shown in different studies^35^.

In accordance with our findings, a recent publication by Han *et al*.^36^ identified other members of the DPP4 family, DPP8 and DPP9, as players in adipocyte differentiation. This group used a permanent mouse cell line (3T3-L1), and in contrast to the effects of DPP4 observed in our work, blocking or knockdown of DPP8 and DPP9 inhibited differentiation with involvement of PPARγ.

One strongly induced gene in response to DPP4 knockdown was PPAΡγC1α. This gene, also known as PGC1α, is related to mitochondrial function and it is necessary for brown fat development^26^,^37^. It acts as a master regulator of mitochondrial biogenesis in mammals and therefore participates in energy balance. However, other genes characteristic for brown or “beige” fat cells, such as UCP1^37^,^38^, were not increased.

In line with previous studies, we detected increased DPP4 protein expression and release in a maturation-dependent way^18^,^39^. This was also observed during monocyte differentiation to dendritic cells^14^. However, in our study adipocyte maturation predominantly affected DPP4 liberation; DPP4 expression changed only to a minor extent. This is in contrast to findings by Das *et al*.^39^ in the mouse cell line 3T3-L1 where a strong increase in DPP4 protein expression during adipocyte differentiation was observed. We could hardly measure a DPP4 release in the supernatants from preadipocytes but observed a marked increase during adipogenesis, pointing to DPP4 release as an important function of mature adipocytes. This is in line with the recently recognized role of the adipose tissue as an endocrine organ^21^. The mechanism underlying increasing DPP4 release during maturation is not clear, and as it is not yet established which factors trigger DPP4 release. Another prominent adipokine, leptin, becomes released in dependence of the adipocyte triglyceride content. No such dependency was observed for DPP4.

Taken together, DPP4 was found to be highly expressed in adipose cells, and its knockdown contributes to differentiation of human preadipocytes, obviously at an early stage and in a PPARγ-independent way. In preadipocytes DPP4 appears to play a different role than in mature adipocytes where it becomes released and thereby can influence glucose metabolism via incretin (e.g. GLP1) cleavage.

## Methods

### Cell culture

Primary human preadipocytes from subcutaneous adipose tissue were commercially obtained from PromoCell (Heidelberg, Germany) and Zen-Bio (Durham, NC, USA). They were cultured according to the manufacturer’s protocol and using media obtained from PromoCell. Differentiation was induced at confluent stages (Day 0) by a medium containing d-biotin (8 μg/ml), dexamethasone (400 ng/ml), 3-isobutyl-1-methylxanthin (44 μg/ml), insulin (0.5 μg/ml), L-thyroxine (9 ng/ml) and pioglitazone (4 μg/ml) for 3 days, followed by cultivation in d-biotin (8 μg/ml), dexamethasone (400 ng/ml), insulin (0.5 μg/ml), 3% FCS and pioglitazone (4 μg/ml) until termination of the experiment. Cells from 5 female subjects (aged 28–58) were used for the experiments, with at least two different donors for key experiments.

### Lentiviral knockdown

Stable knockdown (KD) of the DPP4 or PPARγ expression was achieved by lentiviral shRNA constructs directed against DPP4 or PPARγ. Lentiviral plasmid vectors containing the shRNA constructs (pLKO.1-U6-sh-NM935-PGK-Puro, pLKO.1-U6-sh-NM712-PGK-Puro and the controls pLKO.1-U6-sh-ctl-PGK-Puro und pLKO.1-CMV-GFP-PGK-Puro) were obtained from Sigma Life Science (St. Louis, MO, USA). Generation of lentiviral particles was performed as described previously^40^. In brief, human embryonic kidney (HEK293T, ATCC, Wesel, Germany) packaging cells were seeded in Dulbecco’s Modified Eagle Medium (DMEM; Gibco, Darmstadt, Germany) on Poly-L-lysin coated 150 mm^2^ dishes.

For transduction, human primary preadipocytes were seeded with a confluence of 30–40%. Within 24 h, cell medium was renewed containing polybrene (3 μg/ml, Santa Cruz Biotechnology, Santa Cruz, CA, USA) and 150 ng (per 6 well) or 20–35 ng reverse transcriptase (per 24 well) virus particles. The GFP expression vector pLKO.1-CMV-GFP-PGK-Puro was used to optimize the transduction protocol, and pLKO.1-U6-sh-ctl-PGK-Puro (“sh-control” or “SHc” for short) was included in each experiment to correct for effects due to the infection process itself. In order to avoid overgrowth by residual untransduced cells in longer-term experiments (>4 days), a selection step with 5 μg/ml puromycin for 72 hours (Santa Cruz Biotechnology, Santa Cruz, CA, USA) was performed starting 3 days after infection.

### RNA Extraction and Real-Time PCR

Cells were harvested with lysis buffer from the RNeasy Total RNA Extraction Kit (Qiagen, Hilden, Germany), and RNA was isolated according to the manufacturer’s protocol. Complementary DNA was synthesized using Reverse Transcriptase Kit (Roche, Mannheim, Germany). Real-Time PCR reactions were performed with the LightCycler SYBR Green Master mix (Roche, Mannheim, Germany) on a Roche LightCycler 480 instrument with denaturation for 10 s at 95 °C and annealing/extension for 105 s at 72 °C. The nucleotide sequences of the PCR primers used are given in Table 2. The expression levels of the target mRNAs were determined by the crossing point (CP) method. The results were corrected for primer efficiency and normalized to the housekeeping gene glyceraldehyde-3-phosphate dehydrogenase (GAPDH).

### DNA Array Hybridization

Cells were harvested with lysis buffer as described above to extract total RNA. Quality of RNA was controlled by the Agilent 2100 Bioanalyzer platform (Agilent Technologies, Santa Clara, CA, USA); RNA Integrity Number (RIN) was between 9.8 and 10. Amplification and labelling (Cy3 and Cy5) were performed using the Low RNA Input Linear Amp Kit (Agilent Technologies, Santa Clara, CA, USA). Hybridization was conducted on Agilent Whole Genome Oligo (60-mer) 4 × 44K microarrays with the Agilent Gene Expression Hybridization Kit. Agilent’s feature extraction Software was used to determine spot intensities and Cy5/Cy3 ratios after background subtraction with ratios displaying the expression level in KD compared to control in a logarithmic scale. Mean fold changes were calculated from four replicate measurements. The heat map was created by Gene Spring Software (Agilent Technologies, Santa Clara, CA, USA).

### Western blotting

Cells were cultured in a 6-well dish and harvested for the experiments or, for signaling experiments, incubated in a serum-free basal medium for 24 h. For analyzing signaling pathways, the latter were activated by treatment with 100 nM insulin (Sigma-Aldrich, Steinheim, Germany) for 10 min. After washing the cells with ice-cold PBS, cellular proteins were extracted with either RIPA lysis buffer (Cell Signaling Technology, Danvers, MA, USA) containing Protease Inhibitor Cocktail (Roche, Mannheim, Germany) and PMSF (Sigma-Aldrich, Steinheim, Germany) for standard Western blotting and signaling experiments or with lysis buffer from Qiagen (Hilden, Germany) for protein fractionation. The latter was performed according to the Qproteome Cell Compartment KIT protocol. For SDS-PAGE, 10–30 μg protein were loaded on a gradient 4–15% polyacrylamide gel in a reducing buffer (Roth, Karlsruhe, Germany). Separated proteins were transferred to a PVDF membrane (Bio-Rad, Munich, Germany) in a tank blotting apparatus (Bio-Rad, Munich, Germany). Blots were blocked in tris-buffered saline containing 0,1% Tween and 5% dried milk protein and incubated over night with anti-DPP4 (1:1000; Abnova, Taipei, Taiwan), anti-PGC1α (1:1000; Cell Signaling Technology, Danvers, MA, USA) or an antibody against the insulin receptor β subunit (CT-3, 1:300, Santa Cruz Biotechnology, Santa Cruz, CA, USA) followed by anti-α-actinin (1:1000; Santa Cruz Biotechnology, Santa Cruz, CA, USA) antibody overnight at 4 °C. HRP-coupled secondary antibodies were from Cell Signaling Technology (Danvers, MA, USA); chemiluminescence signals were detected by chemiluminescent reagent (ECL; Thermo Scientific, Rockford, IL, USA). For signaling experiments, the phosphoproteins were detected by anti-pAkt (Ser473; 1:1000; Cell Signaling Technology, Danvers, MA, USA) or by anti-pERK (Thr202/Tyr204; 1:500; Cell Signaling Technology, Danvers, MA, USA) and secondary anti-mouse antibodies (Dianova, Hamburg, Germany). Furthermore, antibodies against total Akt (1:1000; Cell Signaling Technology, Danvers, MA, USA) or ERK (1:1000; Cell Signaling Technology, Danvers, MA, USA) with secondary anti-rabbit IgG antibodies were used. Quantification of the ECL-developed bands was performed by densitometric analysis with ImageJ software. The optical density was normalized to the loading control.

### Elisa

Release of DPP4 or leptin from human primary adipocytes were detected in the cell culture supernatants by ELISA (R&D Systems, Wiesbaden, Germany). Cells were cultured in either growth or differentiation media. After renewal of media, cells were further incubated for 24 h and finally, cell supernatants were collected for analysis. The assays were performed according to the manufacturer’s protocol and measured in a microplate reader (Dynex, Chantilly, VA, USA) against a standard curve.

### Oil Red O Staining

Preadipocytes were treated with either 10 μM pioglitazone for 10 days or lentiviral particles according to our standard protocol (see respective subsection). For Oil Red O staining, cells were washed in phosphate buffered saline (PBS) and fixed with 4% paraformaldehyde at 4 °C for 1 h. Subsequently, cells were washed again and incubated with 3 mg/ml Oil Red O solution (Sigma, Steinheim, Germany) for 1 h at room temperature. Finally, the staining solution was replaced by aqua dest., and evaluation of the lipid droplets was done by light microscopy (Leica Microsystems, Wetzlar, Germany).

### Immunofluorescence

For detection of DPP4 by immunofluorescence, human preadipocytes were seeded on poly-L-lysin coated cover slips. Cells were washed in PBS and fixed with 4% paraformaldehyde at 4 °C for 1 h. Blocking was done in a 0,1% BSA solution (Sigma-Aldrich, Steinheim, Germany) for 1 h. The incubation with primary antibody anti-DPP4 (Abnova, Taipei, Taiwan) was performed in a 1:50 dilution overnight. Secondary antibody (Life Technologies, Darmstadt, Germany) was incubated in a 1:200 dilution for 90 min. in the dark. Cells were counterstained with Texas Red Phalloidin (Life Technologies, Darmstadt, Germany) and the fluorescence signals were visualized on a laserscanning microscope (Zeiss, Jena, Germany) in a z-stack analysis.

### Lipolysis

Mature adipocytes were washed in PBS and incubated in serum free medium for 24 h. Then medium containing 10 μM forskolin (Sigma-Aldrich, Steinheim, Germany) or vehicle DMSO was added. After 24 h, cell culture supernatants were collected for analysis of leptin and DPP4 by ELISA. For confirmation of lipolysis, free glycerol was determined in the supernatant. To this end, free glycerol reagent (Sigma-Aldrich, Steinheim, Germany) was added to the supernatants and incubated for 3 h at room temperature in the dark. Finally, absorption at 540 nm was measured in an ELISA reader. A standard curve was obtained with ascending dilutions of glycerol in assay buffer.

### Statistical analysis

All data are represented as means ± SEM. Determination of significant differences was done by Student *t* test, linear regression analysis or between groups by ANOVA followed by Dunnett post-test. Samples were normalized against controls by inclusion of a baseline correction. Statistical analyses were performed by Graph Pad Prism Software (Graph Pad Software, La Jolla, CA, USA).

## Additional Information

**How to cite this article**: Zilleßen, P. *et al*. Metabolic role of dipeptidyl peptidase 4 (DPP4) in primary human (pre)adipocytes. *Sci. Rep.* **6**, 23074; doi: 10.1038/srep23074 (2016).

**Accession codes**: Gene chip data shown in Fig. 2C were submitted to GEO database; the accession number is GSE75328.

## Supplementary Material

Supplementary Information

## Figures and Tables

**Table 1:** Effects of DPP4 gene expression knockdown in human primary preadipocytes.

Abbreviation, accession	Gene	log(ratio) mean, SEM	Mean fold change	Description
**A. Lipid metabolism**				
PLIN5 NM_001013706	Homo sapiens lipid storage droplet protein 5	1,41 ± 0,06	25,43	coats lipid droplets
PDK4 NM_002612	Homo sapiens pyruvate dehydrogenase kinase	1,37 ± 0,08	23,48	involved in glucose metabolism
CEBPE NM_001805	Homo sapiens CCAAT/enhancer binding protein	1,31 ± 0,33	20,25	transcription factor
PLIN NM_002666	Homo sapiens perilipin	1,15 ± 0,21	14,20	coats lipid droplets
FABP4 NM_001442	fatty acid binding protein 4	1,15 ± 0,12	14,19	maybe involved in fatty acid metabolism
ACSM5 NM_017888	Homo sapiens acyl-CoA synthetase medium-chain family member 5	1,11 ± 0,02	12,85	involved in fatty acid metabolism
APOE NM_000041	Homo sapiens apolipoprotein E	1,10 ± 0,07	12,66	involved in fatty acid metabolism
KLF15 NM_014079	Homo sapiens Kruppel-like factor 15	0,75 ± 0.09	5.65	transcription factor
PPARGC1A NM_013261	Homo sapiens peroxisome proliferator-activated receptor gamma	0,70 ± 0,09	4,99	involved in energy metabolism
KLF2 NM_016270	Homo sapiens Kruppel-like factor 2	−0,70 ± 0.04	−5.01	transcription factor
FADS1 NM_013402	fatty acid desaturase 1	−1,03 ± 0,02	−10,83	involved in fatty acid metabolism
FADS2 NM_004265	fatty acid desaturase 2	−1,14 ± 0,05	−13,87	involved in fatty acid metabolism
**B. Proliferation**				
FGFR1 NM_023110	Homo sapiens fibroblast growth factor receptor 1	−0,31 ± 0,06	−2,06	growth factor receptor
TIMP1 NM_003254	Homo sapiens TIMP metallopeptidase inhibitor 1	−0,57 ± 0,03	−3,70	involved in cell proliferation, inhibitors of the matrix metalloproteinases
CAPNS1 NM_001749	Homo sapiens calpain, small subunit 1	−0,59 ± 0,04	−3,91	involved in proliferation and adhesion
FGF7 NM_002009	fibroblast growth factor 7 (keratinocyte growth factor)	−0,78 ± 0,07	−6,02	growth factor
PDGFRL NM_006207	Homo sapiens platelet-derived growth factor receptor-like	−0,94 ± 0,02	−8,66	growth factor receptor-like
SULF1 NM_015170	Homo sapiens sulfatase 1	-1,67 ± 0,02	-47,18	coreceptor for growth factors and cytokines
**C. Structural genes / cell-cell contact**				
HPSE NM_006665	Homo sapiens heparanase	1,35 ± 0,02	22,32	component of the membrane and extracellular matrix
HAS1 NM_001523	Homo sapiens hyaluronan synthase 1	1,22 ± 0,11	16,57	component of the extracellular matrix
ITGB3 NM_000212	Homo sapiens integrin, beta 3	0,36 ± 0,04	2,30	involved in cell adhesion
COL4A1 NM_001845	Homo sapiens collagen, type IV, alpha 1	0,32 ± 0,08	2,07	component of the membrane
COL13A1 NM_080801	Homo sapiens collagen, type XIII, alpha 1	−0,37 ± 0,10	−2,36	component of the membrane
COL1A1 NM_000088	Homo sapiens collagen, type I, alpha 1	−0,45 ± 0,10	−2,82	component of connective tissue
RAC1 NM_018890	Homo sapiens ras-related C3 botulinum toxin substrate 1	-0,61 ± 0,04	−4,11	maybe involved in cell growth, cytoskeletal reorganization
KRT19 NM_002276	Homo sapiens keratin 19	−0,72 ± 0,03	−5,21	involved in structural integrity
SPARC NM_003118	Homo sapiens secreted protein	−1,11 ± 0,06	−12,92	involved in extracellular matrix synthesis
**D. Cell migration**				
RARRES2 NM_002889	Homo sapiens retinoic acid receptor responder 2	1,44 ± 0,07	27,39	chemotactic protein
ANGPT1 NM_001146	Homo sapiens angiopoietin 1	0,42 ± 0,06	2,65	involved in vascular development and angiogenesis
ILK NM_001014795	Homo sapiens integrin-linked kinase (ILK)	−0,53 ± 0,05	−3,37	involved in integrin-mediated signal transduction
VEGFB NM_003377	Homo sapiens vascular endothelial growth factor B	−0,54 ± 0,06	−3,49	vascular endothelial growth factor
PDGFRA NM_006206	Homo sapiens platelet-derived growth factor receptor, alpha polypeptide	−0,98 ± 0,03	−9,57	cell surface receptor
ACVRL1 NM_000020	Homo sapiens activin A receptor type II-like 1	−1,37 ± 0,01	−23,27	cell-surface receptor

Whole genome oligo microarray (Agilent) analysis was used to investigate changes in the expression profile of human primary preadipocytes transduced with shRNA against DPP4. Functional clusters of the most strongly altered genes are displayed, with the data represent the log (ratio) and mean fold change ± SEM of the gene expression after knockdown of DPP4 vs. control. Mean data result of 4 replicate measurements.

**Table 2:** Nucleotide sequences of the PCR primers used.

Abbreviation	Accession number	Upstream primer	Downstream primer
GAPDH	NM_002046	TCCTGTTCGACAGTCAGCCGCAT	TGAAGACGCCAGTGGACTCCACG
DPP4	NM_001935	CTCCAGAAGACAACCTTGACCATTACAGAA	TCATCATCATCTTGACAGTGCAGTTTTGAG
FABP4	NM_001442	TCATACTGGGCCAGGAATTTGACGA	ATGCGAACTTCAGTCCAGGTCAACG
ACSM5	NM_017888	TGGAGCTTTGAGGAGCTGGGGAAG	CCGGTGGTTCCGCTGGTAAAGTAGA
APOE	NM_000041	CAGGCAGGAAGATGAAGGTTCTGTGG	CGCCACCGGGGTCAGTTGTT
PPARγC1α	NM_013261	AGGGACGTCTTTGTGGCTTTTGCTG	ATGGCGTGGGACATGTGCAACC
PDK4	NM_002612	TCACAGACAGGAAACCCAAGCCACA	CCGTAACCAAAACCAGCCAAAGGAG
PLIN1	NM_002666	GCCTTGGGCAGCATTGAGAAGGT	CCTCTCCCTCCGTGTCTGTCTGGT
SULF1	NM_015170	AGCGTGGAAGGACCATAAGGCATACA	TCCAAAAAGCCAGTAGCAAACTCACAGAA
PDGFRL	NM_006207	AAGGTCTGGCTGCTGCTTGGTCTTC	ACCTGTGTCTGCCGAGGTGGAGTT
FGF7	NM_002009	AACTGTTCCAGCCCTGAGCGACAC	GCAACAAACATTTCCCCTCCGTTG
