# Supplementary material for: Metabolic role of dipeptidyl peptidase 4 (DPP4) in primary human (pre)adipocytes
Source: Sci Rep. 2016 Mar 17;6:23074. doi: 10.1038/srep23074 (PMC4794806; doi:10.1038/srep23074)
Supplement: Supplementary Information [file srep23074-s1.doc]

**Metabolic role of dipeptidyl peptidase 4 (DPP4) in primary human (pre)adipocytes**

Pia Zilleßen, Jennifer Celner, Anita Kretschmann, Alexander Pfeifer, Kurt Racké, Peter Mayer

**Supplementary Figures**


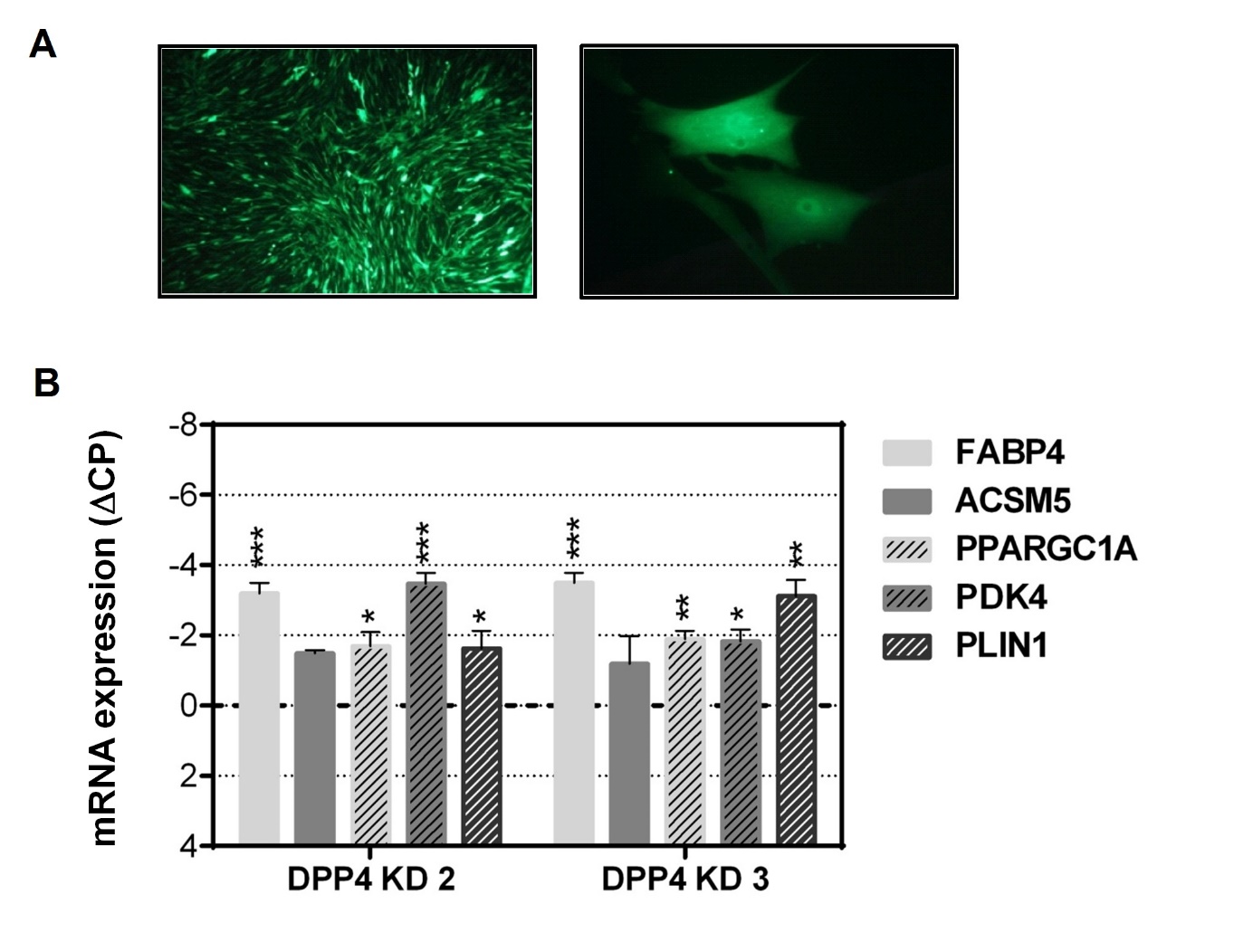


**Figure S1.** Verification of cell transduction and DPP4 knockdown. Part **A**: Successful infection of the preadipocytes was verified by expression of GFP constructs, visualized by fluorescence microscopy. Two different magnifications are shown. Part **B**: In order to verify that the observed effects were indeed due to DDP4 knockdown and not due to any unspecific sequels of transduction, we employed two alternative shRNA constructs. All three shRNA constructs had a different nucleotide sequence and were targeted to different sites on the DPP4 mRNA. Expression of representative genes was measured by RT PCR after knockdown of DPP4 using the two alternative shRNA constructs, labeled “DPP4 KD 2” and “DPP4 KD 3”. Data are normalized to control and are displayed as means + SEM of ≥ 3 replicates. Statistical significance is indicated as follows: *p < 0.05; **p < 0.01; ***p < 0.001.


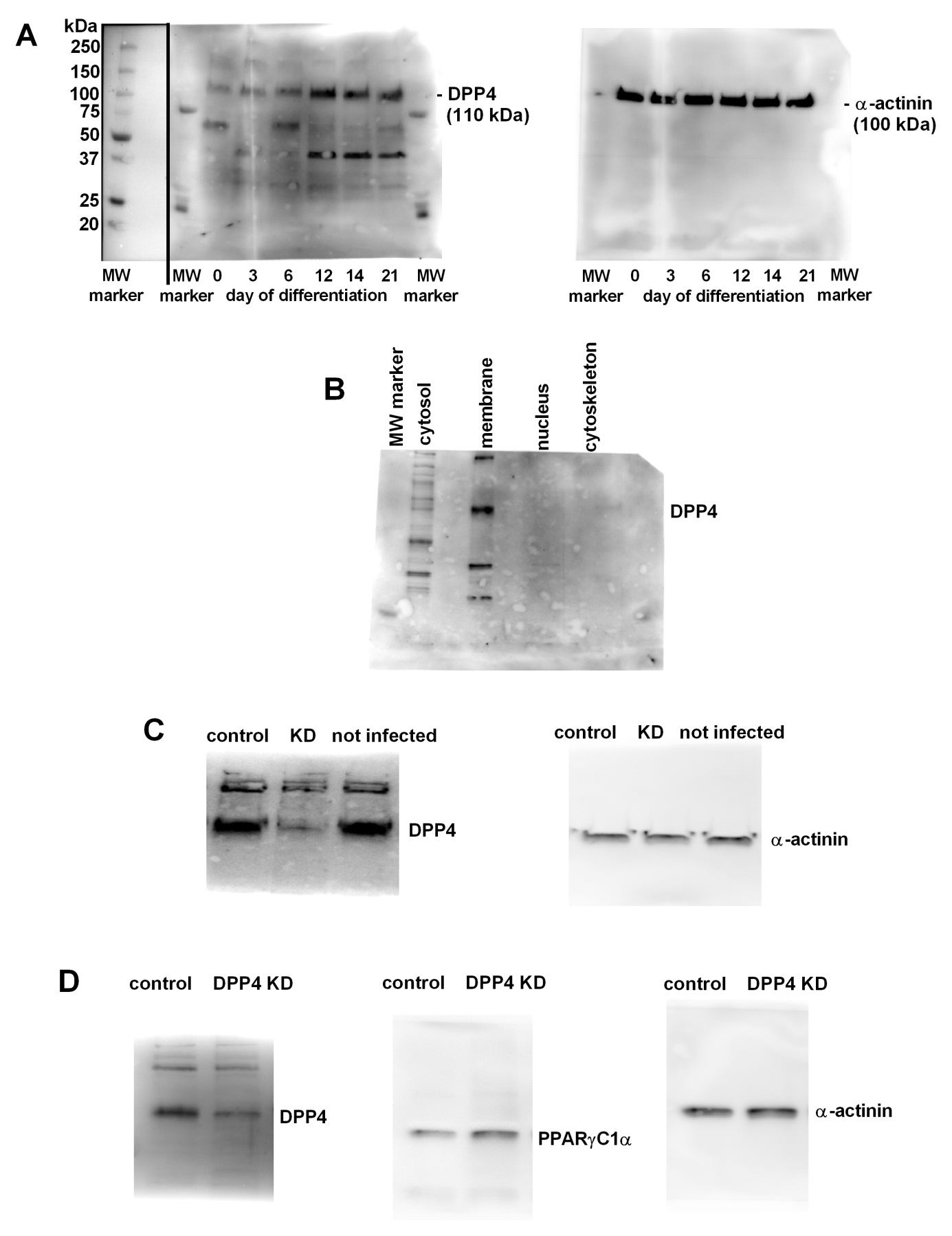


**Figure S2.** Raw data of the Western blotting experiments presented in the main Figures 1 and 2. The bands on the blotting membranes were visualized by chemiluminescence using commercial reagent kits (see Methods section for further details). In all cases the lanes are displayed over the entire length of the blotting membrane. The blotting membrane did not always cover the whole gel in order to save material. Test and control antibodies were always incubated with the same blotting membrane; the membrane was stripped before incubation with the next antibody. Loading with sequential antibodies is indicated in the figure by panel arrangement from left to right. A molecular weight (MW) marker was loaded on each gel and visualized separately, representatively shown the left panel of Part **A**. In Parts **C** and **D** of the figure not all lanes are shown because for the samples of interest only part of the gel was used. The remaining slots were filled with pilot samples unrelated to this work. Part **A** corresponds to Main Figure 1C. The blotting membrane was first incubated with anti-DPP4 and, after stripping, with anti-α-actinin as loading control. Part **B** corresponds to Main Figure 1F; loading control with α-actinin was not possible in this case because this protein is not present in all cell fractions. Part **C** corresponds to Main Figure 2B, and Part **D** corresponds to Main Figure 2E.

**
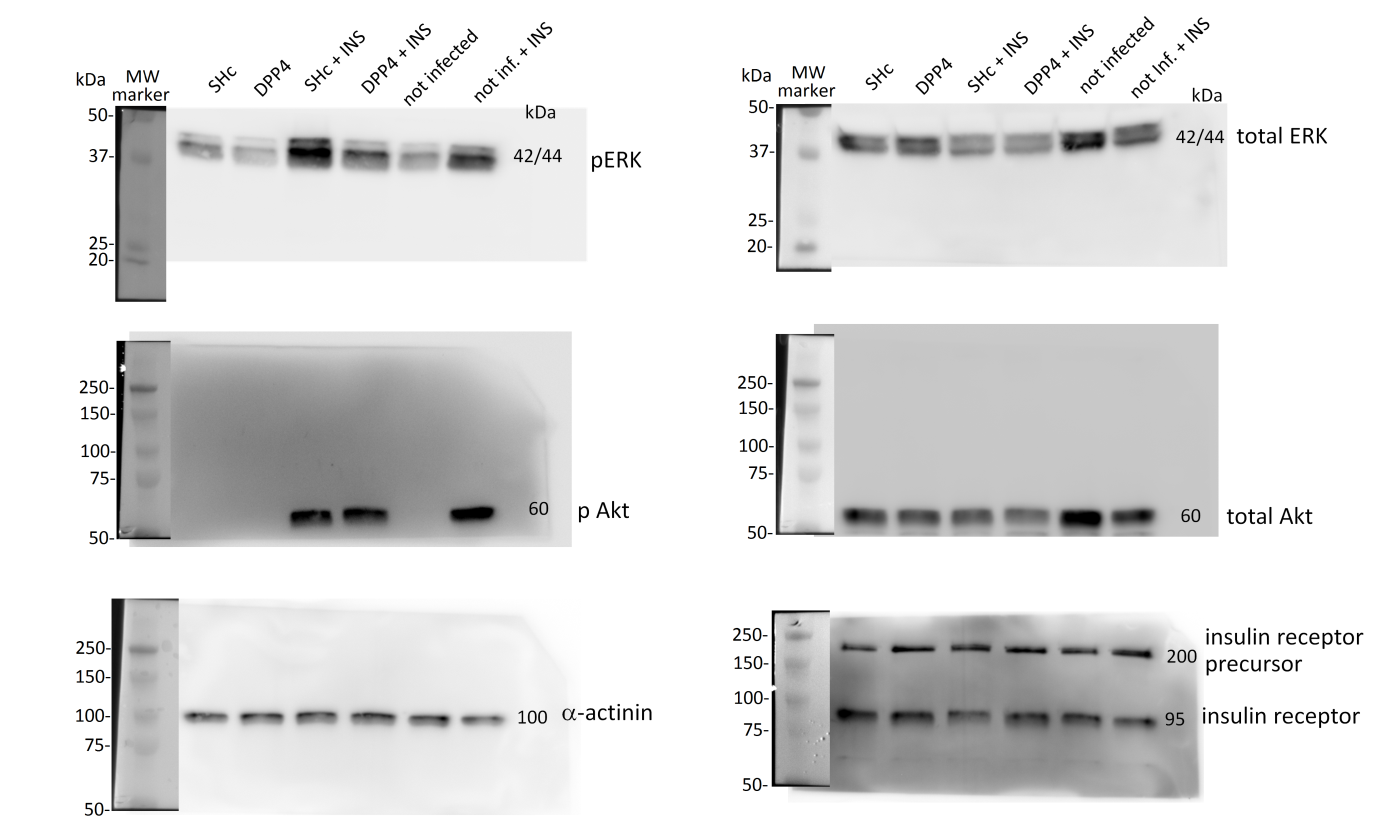
**

**Figure S3.** Raw data of the Western blotting experiments presented in Main Figure 5. With the polyacrylamide gels used, Akt (60 kDa) and ERK (42/42 kDa) were well separated so that blotting membranes could be cut at 50 kDa after protein transfer. Thus, (p)Akt and (p)ERK could be detected simultaneously in one experiment without the need of membrane stripping, which would reduce quality. The upper half was then re-blotted after stripping with antibodies against α-actinin and insulin receptor (see Methods section for more information on the antibodies used). Hence, for the blots of pAkt, total Akt, α-actinin and insulin receptor the upper half of the membrane is shown and for pERK and total ERK the lower half.

Detection of the insulin receptor was done with antibody CT-3 which is directed against the beta subunit of the receptor (lower bands in the respective panel). The insulin receptor precursor, i.e. the receptor protein before separation of the alpha and beta subunit by proteolytic cleavage, is also detected by this antibody (upper bands in the panel).
